# The association between serum S100β levels and prognosis in acute stroke patients after intravenous thrombolysis: a multicenter prospective cohort study

**DOI:** 10.1186/s12916-024-03517-6

**Published:** 2024-10-03

**Authors:** Yang Qu, Hang Jin, Reziya Abuduxukuer, Shuang Qi, Xiang-Kun Si, Peng Zhang, Ke-Jia Zhang, Si-Ji Wang, Xiang-Yu Zheng, Yu Zhang, Jian-Hua Gao, Xian-Kun Zhang, Xiao-Dong Liu, Chun-Ying Li, Guang-Cai Li, Junmin Wang, Huimin Jin, Ying He, Ligang Jiang, Liang Liu, Yongfei Jiang, Rui-Hong Teng, Yan Jia, Bai-Jing Zhang, Zhibo Chen, Yingbin Qi, Xiuping Liu, Song Li, Xin Sun, Thanh N. Nguyen, Yi Yang, Zhen-Ni Guo, on behalf of the Biomarkers of Brain Injury Investigator Study Group, Lijuan Wang, Yumei Chen, Yang Zheng, Zhi-Mei Yuan, Dongsheng Ju, Yun-Fei Ba, Jinhua Chen, Jiliang Gu, Anying Wang, Li-Li Zhao, Chen-Peng Dong, Li Liu, Zhong-Rui Pei, Shuang Yu, Xue Liu, Chun-Li Jiang, Ling He, Sun-Juan Zhang

**Affiliations:** 1 Stroke Center, Department of Neurology, the First Hospital of Jilin University, Changchun, China; 2 Neuroscience Research Center, Department of Neurology, the First Hospital of Jilin University, Changchun, China; 3 Department of Neurology, the First Hospital of Jilin University, Changchun, China; 4 Department of Neurology, Songyuan Central Hospital, Songyuan, China; 5 Department of Neurology, Jilin Neuropsychiatric Hospital, Siping, China; 6 Stroke Center, Department of Neurology, Siping Central People’s Hospital, Siping, China; 7 Department of Neurosurgery, Tonghua City Vascular Disease Hospital and Dongchang District People’s Hospital, Tonghua, China; 8 Department of Neurology, Songyuan Jilin Oilfield Hospital, Songyuan, China; 9 Stroke Center, Department of Neurology, Dehuishi People’s Hospital, Changchun, China; 10 Department of Neurology, Affiliated Hospital of Jilin Medical College, Jilin, China; 11 Department of Neurology, Songyuan Hospital of Integrated Traditional Chinese and Western Medicine, Songyuan, China; 12 Stroke Center, Department of Neurology, Qianguoerros Mongolian Autonomous County Hospital, Songyuan, China; 13 Department of Neurology, Jilin City Hospital of Chemical Industry, Jilin, China; 14 Department of Neurology, Changchun People’s Hospital, Changchun, China; 15 Department of Neurology, Changchun Second Hospital, Changchun, China; 16 Department of Neurology, Dongliao First People’s Hospital, Liaoyuan, China; 17 Department of Neurology, Jilin People’s Hospital, Jilin, China; 18 Department of Neurology, Jilin Province People’s Hospital, Changchun, China; 19 Stroke Center, Department of Neurology, Jilin Central General Hospital, Jilin, China; 20 Boston Medical Center Neurology, Radiology, Boston Medical Center, Boston University Chobanian and Avedisian School of Medicine, Boston, USA

**Keywords:** S100β, Intravenous thrombolysis, Acute ischemic stroke, Outcome, Astroglial injury

## Abstract

**Background:**

S100β is a biomarker of astroglial damage, the level of which is significantly increased following brain injury. However, the characteristics of S100β and its association with prognosis in patients with acute ischemic stroke following intravenous thrombolysis (IVT) remain unclear.

**Methods:**

Patients in this multicenter prospective cohort study were prospectively and consecutively recruited from 16 centers. Serum S100β levels were measured 24 h after IVT. National Institutes of Health Stroke Scale (NIHSS) and hemorrhagic transformation (HT) were measured simultaneously. NIHSS at 7 days after stroke, final infarct volume, and modified Rankin Scale (mRS) scores at 90 days were also collected. An mRS score ≥ 2 at 90 days was defined as an unfavorable outcome.

**Results:**

A total of 1072 patients were included in the analysis. The highest S100β levels (> 0.20 ng/mL) correlated independently with HT and higher NIHSS at 24 h, higher NIHSS at 7 days, larger final infarct volume, and unfavorable outcome at 3 months. The patients were divided into two groups based on dominant and non-dominant stroke hemispheres. The highest S100β level was similarly associated with the infarct volume in patients with stroke in either hemisphere (dominant: *β* 36.853, 95% confidence interval (CI) 22.659–51.048, *P* < 0.001; non-dominant: *β* 23.645, 95% CI 10.774–36.516, *P* = 0.007). However, serum S100β levels at 24 h were more strongly associated with NIHSS scores at 24 h and 3-month unfavorable outcome in patients with dominant hemisphere stroke (NIHSS: *β* 3.470, 95% CI 2.392–4.548, *P* < 0.001; 3-month outcome: odds ratio (OR) 5.436, 95% CI 2.936–10.064, *P* < 0.001) than in those with non-dominant hemisphere stroke (NIHSS: *β* 0.326, 95% CI  − 0.735–1.387, *P* = 0.547; 3-month outcome: OR 0.882, 95% CI 0.538–1.445, *P* = 0.619). The association of S100β levels and HT was not significant in either stroke lateralization group.

**Conclusions:**

Serum S100β levels 24 h after IVT were independently associated with HT, infarct volume, and prognosis in patients with IVT, which suggests the application value of serum S100β in judging the degree of disease and predicting prognosis.

**Supplementary Information:**

The online version contains supplementary material available at 10.1186/s12916-024-03517-6.

## Background

The global burden of disability caused by stroke has increased in recent years [1]. Intravenous thrombolysis (IVT) has the highest level of evidence for the treatment of acute disabling ischemic stroke [2]; however, approximately half of these patients do not achieve favorable outcomes following IVT [3]. Timely and accurate assessment of patients’ conditions can help neurologists formulate treatment strategies and adjust therapeutic regimes.

S100β is a calcium-binding protein mainly concentrated in astrocytes, and its elevation reflects astrocyte injury [4, 5]. Due to damage to brain tissue and the blood–brain barrier in patients with stroke, S100β is released and enters the bloodstream, resulting in abnormally high levels of S100β in the blood [6]. Previous studies found that serum S100β concentrations were highly correlated with final infarct volume in patients with ischemic stroke [7, 8]. Moreover, Branco et al. demonstrated that S100β levels in peripheral blood at 48 h post-stroke were associated with functional outcome at 3 months [9], which suggests S100β as a prognosis marker for patients with stroke. In patients following IVT, thrombolytic drugs may decrease the final infarct size but increase the breakdown of the blood–brain barrier [10]. Therefore, the characteristics of serum S100β levels in these patients and their relationship with prognosis are unique. Further investigation with a large patient sample size remains warranted.

In the present study, we collected blood samples 24 h after IVT from patients at 16 centers in northeast China to (a) test the serum S100β levels in patients 24 h after IVT; (b) explore the relationship between serum S100β levels and hemorrhagic transformation (HT); and (c) explore the association between serum S100β levels and final infarct volume, National Institutes of Health Stroke Scale (NIHSS), and modified Rankin Scale (mRS) at 90 days in these patients.

## Methods

This study was conducted using serum samples from a prospective cohort of patients who underwent IVT. This study was approved by the Ethics Committee of the First Hospital of Jilin University (2015–156). Written informed consent was obtained from all participants, and these patients had the right to withdraw from the study at any point.

### Participants

**Fig. 1:**
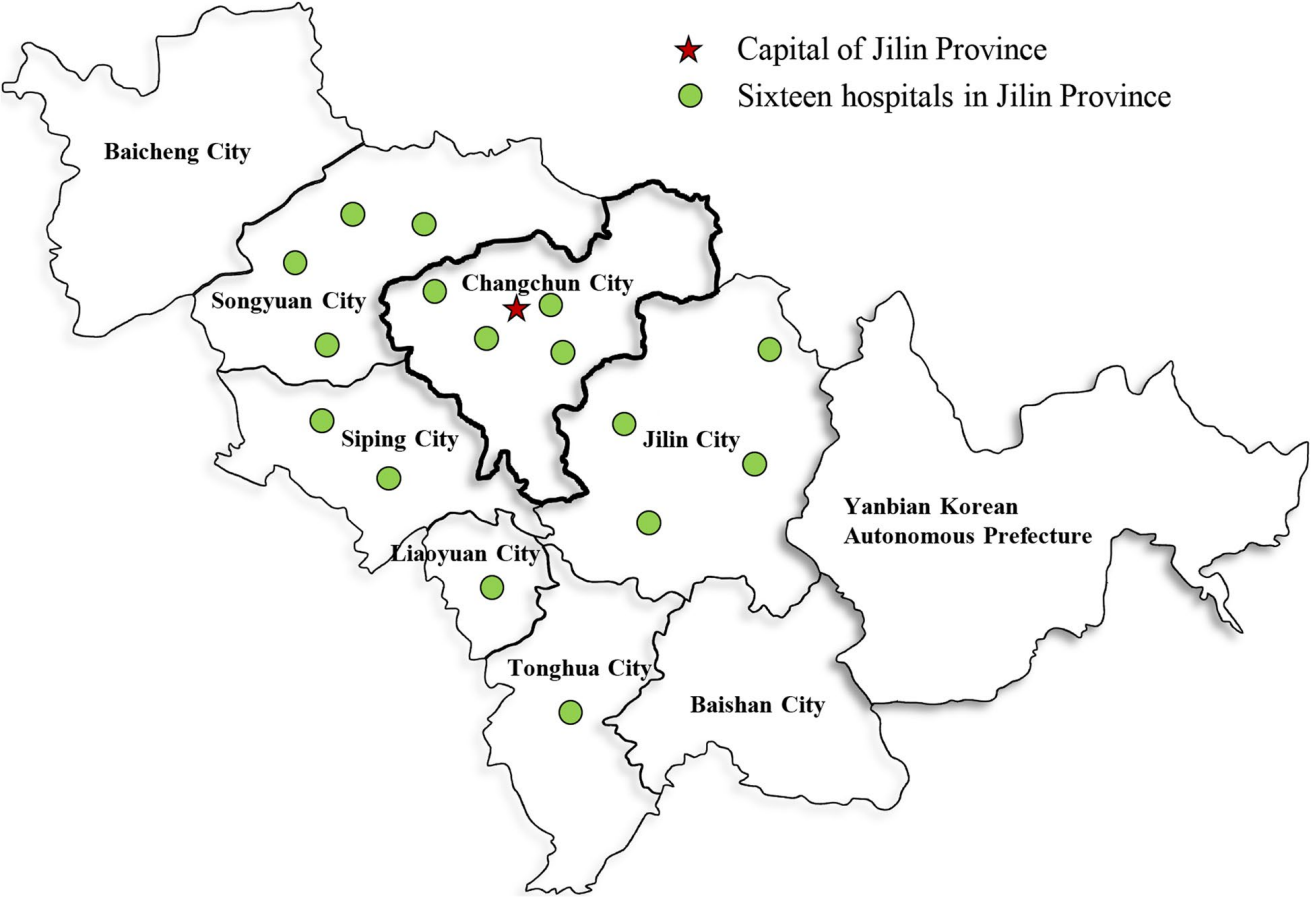
Geographical distribution of the hospitals

In this prospective study, we enrolled consecutive patients who met the eligibility for IVT and underwent alteplase IVT, and additionally met the following inclusion criteria between September 2016 and April 2023 at the First Hospital of Jilin University and between September 2021 to January 2022 at the other 15 hospitals in northeast China (Fig. 1, Additional file 1: Table S1). The additional inclusion criteria were (a) age > 18 years, (b) underwent standard alteplase treatment (0.9 mg/kg), and (c) had been functioning independently before the stroke (mRS, 0–1; range, 0 (no symptoms) to 6 (death)).

### Clinical parameters

The following baseline clinical variables were collected: demographic features, vascular risk factors, and clinical data. The demographic features included name, age, and sex. Vascular risk factors included cigarette smoking, alcohol consumption, hypertension, diabetes mellitus, dyslipidemia, hyperhomocysteinemia, previous ischemic stroke, and coronary heart disease [11, 12]. Clinical information included blood pressure, heart rate, blood glucose, onset-to-alteplase bolus time, stroke severity, infarct location, stroke subtypes, and bridging therapy. Blood pressure and heart rate were measured on admission. Blood glucose levels were measured the morning following admission after overnight fasting. Stroke severity was assessed using the NIHSS at admission, 24 h, and 7 days after IVT via physical examination [13]. Stoke subtypes were classified according to the Trial of Org 10,172 in the Acute Stroke Treatment (TOAST) classification [14]. In addition, the mRS score at 90 days was recorded via telephone interviews with each patient or next of kin. A mRS score ≤ 1 was defined as a favorable outcome, and an unfavorable outcome was defined as a mRS score > 1 90 days after stroke onset. Any cause of death within 90 days was also recorded. HT was defined as any visible hemorrhage on brain computed tomography 24 h after IVT and classified as hemorrhagic infarction type 1 (HI1), hemorrhagic infarction type 2 (HI2), parenchymal hematoma type 1 (PH1), parenchymal hematoma type 2 (PH2), and remote parenchymal hematoma (rPH) according to the European Collaborative Acute Stroke Study (ECASS) [15]. Infarct volume was calculated on brain diffusion-weighted magnetic resonance imaging obtained 3–7 days after stroke onset, using open-source 3D Slicer software, version 5.3.0 [16]. The mRS and imaging parameters were assessed by examiners blinded to the clinical parameters and serum biomarker levels.

### Blood sampling and serum S100β measurement

Venous blood samples were drawn from the cubital vein of each patient 24 h after IVT, centrifuged to obtain serum, transferred to cryovials, and kept frozen at − 80 °C in the Department of Biobank, Division of Clinical Research, the First Hospital of Jilin University until analysis within 2 h following collection. The determination of the level of biomarker S100β was performed at the laboratory of the Stroke Center, the First Hospital of Jilin University, via an automated magnetic particle-based chemiluminescent enzyme immunoassay analyzing system (MS-Fast/Aceso 80A, Sophonix, Beijing, China) as previously reported [17]. The test reagent is individually pre-loaded in a single cartridge. The entire detection procedure is completely automated and requires only 28 min. Briefly, the procedure is as follows: S100β proteins were combined with an alkaline phosphatase-labeled antibody and a biotinylated antibody to form a sandwich structure. An excess of streptavidin-coated magnetic particles was introduced and combined with the biotinylated antibody to form a complex. The complexes were enriched by a magnetic field, thereby enhancing signal sensitivity and allowing them to be captured. After washing, a luminescent substrate was added and cleaved by the enzyme in the complex to form an unstable excited-state intermediate. When the excited-state intermediate returns to its ground state, it emits photons. The light intensity was detected using a photomultiplier tube and converted into the concentration of the test sample. Serum samples were processed and assayed by laboratory technicians blinded to all clinical parameters, and the assay results were not provided to the healthcare providers. The lower limit of quantification was 0.05 ng/mL, while the upper limit was 10 ng/mL. The coefficient of variation was < 8.0%. Concentrations of S100β below the limit of quantification were analyzed as 0.05 ng/mL, whereas those above as 10 ng/mL.

### Statistical analysis

Statistical analyses were performed using IBM SPSS Statistics (version 26.0; Statistical Package for the Social Sciences, Armonk, NY, USA). The distribution of data was assessed using a one-sample Kolmogorov–Smirnov test. Continuous variables are expressed as means and standard deviations or medians and interquartile ranges, according to a normal or skewed distribution. Categorical variables are described as frequencies and percentages. Study participants were divided into three groups according to tertiles of S100β levels. Clinical variables and outcome parameters were compared among the three groups using one-way analysis of variance, *χ* ^2^ test, Fisher’s exact test, or the Kruskal–Wallis test, based on the measurement level. Comparisons were corrected post hoc using Bonferroni adjustment. S100β values between the two independent groups were compared using Mann–Whitney *U* test. S100β values between patients with different types of HT were compared using the Kruskal–Wallis test.

To explore the correlation between S100β levels and HT, NIHSS, infarct volume, and 3-month outcome, univariable and multivariable linear regression and binary logistic regression were used. Five models were applied in the sensitivity analysis: Model 1 was unadjusted; Model 2 was adjusted for age and sex; Model 3 was adjusted for age, sex, and vascular risk factors (including cigarette smoking, alcohol consumption, hypertension, diabetes mellitus, dyslipidemia, hyperhomocysteinemia, previous ischemic stroke, and coronary heart disease); Model 4 was adjusted for age, sex, vascular risk factors, and clinical data (including blood pressure, heart rate, blood glucose, admission NIHSS score, onset-to-alteplase bolus time, TOAST, infarct location, and bridging therapy); and Model 5 was adjusted using the confounders of Model 4 except for admission NIHSS score.

Subgroup analyses were reported in unadjusted and adjusted models (adjusted for the same prespecified covariates as in the primary analyses) across different stroke lateralizations, and the heterogeneity of the association of S100β with clinical parameters and outcomes was assessed. A two-sided *P* < 0.05 was considered statistically significant.

## Results

### Participant characteristics

Of the 1580 patients who received alteplase IVT, blood samples were collected from 1131; 22 declined to participate in the study during follow-up, 37 were lost to 3-month follow-up, and the remaining 1072 were included in the analysis. The flowchart of the study is shown in Additional file 1: Fig. S1. The characteristics of the patients are listed in Table 1, of whom 478 (44.6%) had favorable outcomes (mRS 0–1) and 52 (4.9%) died due to all causes at 3 months. Computed tomography was available for 1070 patients, of whom 93 (8.7%) had HT 24 h after IVT.

**Table 1:** Clinical characteristics and outcomes

Variables	Total (*n* = 1072)	T1 (S100β ≤ 0.08 ng/mL, *n* = 385)	T2 (0.08 < S100β ≤ 0.20 ng/mL, *n* = 340)	T3 (S100β > 0.20 ng/mL, *n* = 347)	*χ* ^2^/*F*	*P*
**Demographics**						
Age (year)	62.58 ± 11.40	61.91 ± 10.77	62.58 ± 12.02	64.30 ± 11.30	5.995	0.003
Sex (male, *n* (%))	758 (70.7%)	217 (70.4%)	244 (71.8%)	243 (70.0%)	0.279	0.870
**Vascular risk factors**						
Cigarette smoking, *n* (%)	545 (50.8%)	207 (53.8%)	185 (54.4%)	153 (44.1%)	9.376	0.009
Alcohol consumption, *n* (%)	428 (39.9%)	165 (42.9%)	154 (45.3%)	109 (31.4%)	15.951	< 0.001
Hypertension, *n* (%)	542 (50.6%)	190 (49.4%)	162 (47.6%)	190 (54.8%)	3.822	0.148
Diabetes mellitus, *n* (%)	216 (20.1%)	76 (19.7%)	69 (20.3%)	71 (20.5%)	0.065	0.968
Dyslipidemia, *n* (%)	727 (67.8%)	261 (67.8%)	235 (69.1%)	231 (66.6%)	0.511	0.775
Hyperhomocysteinemia, *n* (%)	479 (44.7%)	151 (39.2%)	160 (47.1%)	168 (48.4%)	7.379	0.025
Previous ischemic stroke, *n* (%)	171 (16.0%)	50 (13.0%)	56 (16.5%)	65 (18.7%)	4.593	0.101
Coronary heart disease, *n* (%)	233 (21.7%)	59 (15.3%)	75 (22.1%)	99 (28.5%)	18.740	< 0.001
**Clinical data**						
SBP (mmHg)	155.5 (140–174)	157 (141–180)	154 (140–171)	155 (138–171)	4.915	0.086
DBP (mmHg)	90 (80–86)	91 (80–100)	90 (81–100)	80 (90–100)	2.741	0.254
HR (beats/min)	78 (68–86)	76 (68–84)	78 (69–86)	78 (68–88)	1.475	0.478
Blood glucose (mmol/L)	5.88 (5.05–7.19)	5.50 (4.93–6.85)	5.83 (5.01–7.15)	6.38 (5.36–7.94)	37.234	< 0.001
Admission NIHSS	7 (5–11)	7 (4–10)	7 (5–11)	8 (5–13)	23.191	< 0.001
NIHSS 24 h	4 (2–9)	3 (1–7)	4 (2–8)	6 (3–12)	63.456	< 0.001
Onset-to-alteplase bolus time (min)	178 (139–225)	176 (134.5–221)	177.5 (144–227)	180 (140–230)	2.648	0.266
TOAST					37.411	< 0.001
LAA	376 (35.1%)	111 (28.8%)	128 (37.6%)	137 (39.5%)		
SAO	429 (40.0%)	187 (48.6%)	132 (38.8%)	110 (31.7%)		
CE	101 (9.4%)	25 (6.5%)	25 (7.4%)	51 (14.7%)		
ODC	20 (1.9%)	6 (1.6%)	9 (2.6%)	5 (1.4%)		
UE	146 (13.6%)	56 (14.5%)	46 (13.5%)	44 (12.7%)		
Infarct location					33.709	< 0.001
Cortex	178 (16.6%)	48 (12.5%)	57 (16.8%)	73 (21.0%)		
Subcortex	487 (45.4%)	198 (51.4%)	157 (46.2%)	132 (38.0%)		
Thalamus	61 (5.7%)	26 (6.8%)	19 (5.6%)	16 (4.6%)		
Cerebellum	23 (2.1%)	5 (1.3%)	8 (2.4%)	10 (2.9%)		
Brainstem	102 (9.5%)	46 (11.9%)	32 (9.4%)	24 (6.9%)		
Multiple	221 (20.6%)	62 (16.1%)	67 (19.7%)	92 (26.5%)		
Hemisphere					1.206	0.877
Dominant	493 (46.0%)	170 (44.2%)	160 (47.1%)	163 (47.0%)		
Non-dominant	488 (45.5%)	179 (46.5%)	154 (45.3%)	155 (44.7%)		
Both	91 (8.5%)	36 (9.4%)	26 (7.6%)	29 (8.4%)		
Infarct volume (mL)^c^	2.37 (0.75–9.17)	1.94 (0.63–5.58)	2.02 (0.69–5.82)	3.84 (0.97–37.76)	40.188	< 0.001
Bridging therapy	44 (4.1%)	10 (2.6%)	16 (4.7%)	18 (5.2%)	3.568	0.168
**HT**^a^	93 (8.7%)	19 (4.9%)	20 (5.9%)	54 (15.6%)	31.025	< 0.001
					42.961	< 0.001
HI1	27 (2.5%)	10 (2.6%)	5 (1.5%)	12 (3.5%)		
HI2	21 (2.0%)	0 (0)	6 (1.8%)	15 (4.3%)		
PH1	20 (1.9%)	5 (1.3%)	4 (1.2%)	11 (3.2%)		
PH2	21 (2.0%)	3 (0.8%)	3 (0.9%)	15 (4.3%)		
rPH	4 (0.4%)	1 (0.3%)	2 (0.6%)	1 (0.3%)		
NIHSS at 7 days^b^	3 (1–7)	2 (0–6)	3 (1–6)	5 (2–10)	60.433	< 0.001
Functional long-term prognosis					28.089	< 0.001
mRS ≤ 1 (favorable)	478 (44.6%)	199 (51.7%)	164 (48.2%)	115 (33.1%)		
mRS > 1 (unfavorable)	594 (55.4%)	186 (48.3%)	176 (51.8%)	232 (66.9%)		
mRS					70.856	< 0.001
0	136 (12.7%)	47 (12.2%)	51 (15.0%)	38 (11.0%)		
1	342 (31.9%)	152 (39.5%)	113 (33.2%)	77 (22.2%)		
2	199 (18.6%)	74 (19.2%)	65 (19.1%)	60 (17.3%)		
3	171 (16.0%)	60 (15.6%)	55 (16.2%)	56 (16.1%)		
4	129 (12.0%)	36 (9.4%)	37 (10.9%)	56 (16.1%)		
5	43 (4.0%)	10 (2.6%)	8 (2.4%)	25 (7.2%)		
6 (death)	52 (4.9%)	6 (1.6%)	11 (3.2%)	35 (10.1%)		

*Abbreviations*: *SBP* Systolic blood pressure, *DBP* Diastolic blood pressure, *HR* Heart rate, *NIHSS* National Institutes of Health Stroke Scale, *TOAST* Trial of Org 10,172 in Acute Stroke Treatment classification, *LAA* Large artery atherosclerosis, *SAO* Small artery occlusion, *CE* Cardioembolism, *ODC* Other determined cause, *UE* Undetermined etiology, *HI* Hemorrhagic infarction, *PH* Parenchymal hematoma, *rPH* remote parenchymal hematoma, *mRS* modified Rankin Scale

^a^Computed tomography scans at 24 h were available for 1070 patients (T1 = 385; T2 = 339; T3 = 346). Therefore, 1070 patients were included in the analysis of HT

^b^The NIHSS scores at 7 days were available for 1056 patients because 16 patients died or discharge within 7 days (T1 = 383; T2 = 338; T3 = 335)

^c^Magnetic resonance imaging was available for 986 patients (T1 = 345; T2 = 314; T3 = 327). Therefore, 986 patients were included in the analysis of infarct volume

Participants were divided into three groups according to tertiles of S100β levels. A comparison of the clinical variables and outcome parameters is shown in Table 1. A higher percentage of patients in the highest S100β group had HT and unfavorable outcomes, and patients in the highest S100β group had larger infarct volume, and higher 24-h and 7-day NIHSS scores than those with lower S100β levels.

### Serum S100β levels and HT

Serum S100β levels were significantly higher in patients with HT 24 h after IVT than in those without HT (0.237 (0.100–0.801) ng/mL vs. 0.118 (0.050–0.240) ng/mL, *P* < 0.001). The differences in S100β levels between patients with different types of HT (HI1, HI2, PH1, and PH2) and those without HT were significant (*H* = 35.840, *P* < 0.001). In binary logistic regression analysis, the highest S100β levels (> 0.20 ng/mL) were significantly correlated with HT, and the correlation was similar in the sensitivity analysis (Additional file 1: Table S2). Further, we explored the heterogeneity in stroke lateralization (dominant, non-dominant, and both hemispheres) of S100β in HT. No significant interaction association was found (*P*_interaction_ = 0.821). Therefore, further respective analysis in patients with dominant or non-dominant hemisphere stroke was not conducted.

### Serum S100β levels and infarct volume

**Fig. 2:**
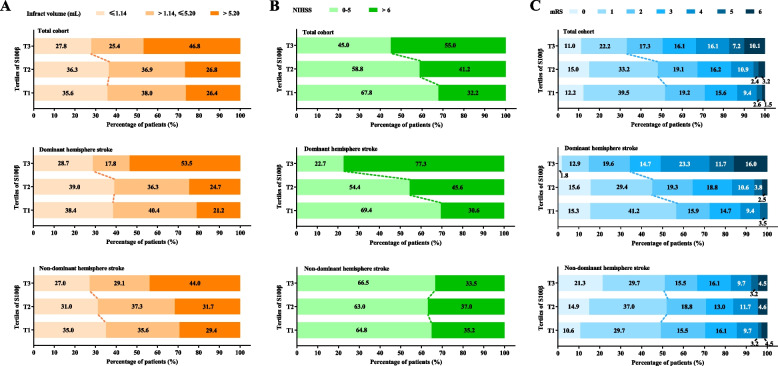
Distribution of **A** final infarct volume, **B** NIHSS at 24 h, and **C** functional outcome assessed by mRS at 90 days according to S100β tertiles in the total cohort and patients with dominant and non-dominant hemisphere stroke. Abbreviations: NIHSS, National Institutes of Health Stroke Scale; mRS, modified Rankin Scale

As shown in Additional file 1: Table S2, the highest S100β levels (> 0.20 ng/mL) were independently correlated with larger infarct volume. In the analysis of heterogeneity in stroke lateralization of S100β in infarct volume, the number of patients with bilateral stroke was small (91 patients, 8.5%); therefore, further univariable and multivariable linear regression analyses were conducted in patients with dominant or non-dominant hemisphere stroke. Although the interaction between stroke lateralization and serum S100β levels in predicting infarct volume was significant (*P*_interaction_ = 0.014), the association between S100β levels and infarct volume was similar between patients with either dominant or non-dominant hemisphere stroke. That is, the highest S100β levels (> 0.20 ng/mL) were independently correlated with larger infarct volume in patients with dominant or non-dominant hemisphere stroke (Table 2 and Additional file 1: Table S3). In addition, the distribution of infarct volume according to S100β levels was similar between patients with dominant and non-dominant hemisphere stroke (Fig. 2A).

**Table 2:** The association of tertiles of S100β with clinical parameters and outcome indicators in patients with different lateralization stroke

	**Model 1**		**Model 2**		**Model 3**		**Model 4**	
	**OR/β (95% CI)**	***P***	**OR/β (95% CI)**	***P***	**OR/β (95% CI)**	***P***	**OR/β (95% CI)**	***P***
**NIHSS 24 h**^a^ (Beta coefficient)								
Dominant hemisphere								
T1	Reference		Reference		Reference		Reference	
T2	1.447 (0.234–2.661)	0.020	1.442 (0.229–2.654)	0.020	1.357 (0.141–2.572)	0.029	0.575 (− 0.406 to 1.556)	0.250
T3	6.594 (5.386–7.802)	< 0.001	6.432 (5.212–7.652)	< 0.001	6.447 (5.203–7.691)	< 0.001	3.470 (2.392–4.548)	< 0.001
*P* for trend	< 0.001		< 0.001		< 0.001		< 0.001	
Non-dominant hemisphere								
T1	Reference		Reference		Reference		Reference	
T2	− 0.052 (− 1.244 to 1.139)	0.931	0.014 (− 1.172 to 1.201)	0.981	− 0.072 (− 1.274 to 1.130)	0.907	− 0.009 (− 1.044 to 1.027)	0.987
T3	0.296 (− 0.894 to 1.485)	0.626	0.315 (− 0.869 to 1.498)	0.602	0.472 (− 0.746 to 1.691)	0.447	0.326 (− 0.735 to 1.387)	0.547
*P* for trend	0.636		0.608		0.462		0.559	
**Infarct volume**^**b**^ (beta coefficient)								
Dominant hemisphere								
T1	Reference		Reference		Reference		Reference	
T2	− 0.258 (− 13.556 to 13.040)	0.970	− 0.010 (− 13.332 to 13.311)	0.999	− 0.468 (− 13.850 to 12.913)	0.945	− 4.211 (− 17.338 to 8.915)	0.529
T3	46.801 (33.741–59.860)	< 0.001	45.939 (32.707–59.170)	< 0.001	46.128 (32.671–59.585)	< 0.001	36.853 (22.659–51.048)	< 0.001
*P* for trend	< 0.001		< 0.001		< 0.001		< 0.001	
Non-dominant hemisphere								
T1	Reference		Reference		Reference		Reference	
T2	5.394 (− 6.950 to 17.738)	0.391	5.306 (− 7.044 to 17.656)	0.399	5.890 (− 6.532 to 18.311)	0.352	7.169 (− 5.206 to 19.544)	0.255
T3	20.783 (8.347–33.220)	< 0.001	20.661 (8.229–33.093)	< 0.001	23.912 (11.114–36.710)	< 0.001	23.645 (10.774–36.516)	< 0.001
*P* for trend	< 0.001		< 0.001		< 0.001		< 0.001	
**mRS 2–6** ^c^ (odds ratio)								
Dominant hemisphere								
T1	Reference		Reference		Reference		Reference	
T2	1.586 (1.027–2.449)	0.038	1.621 (1.046–2.511)	0.031	1.561 (0.997–2.446)	0.052	1.383 (0.849–2.254)	0.193
T3	7.514 (4.428–12.750)	< 0.001	7.160 (4.203–12.199)	< 0.001	8.041 (4.602–14.047)	< 0.001	5.436 (2.936–10.064)	< 0.001
*P* for trend	< 0.001		< 0.001		< 0.001		< 0.001	
Non-dominant hemisphere								
T1	Reference		Reference		Reference		Reference	
T2	0.895 (0.581–1.377)	0.612	0.915 (0.592–1.412)	0.687	0.903 (0.581–1.405)	0.652	0.850 (0.528–1.368)	0.503
T3	0.930 (0.605–1.430)	0.742	0.937 (0.607–1.444)	0.766	0.943 (0.602–1.477)	0.798	0.882 (0.538–1.445)	0.619
*P* for trend	0.963		0.758		0.784		0.597	

The number of patients with both sides of stroke was limited; therefore, the analysis was conducted in patients with dominant or non-dominant hemisphere stroke. The results of Model 5 were the same with those of Models 1–4 and were shown in Additional file 1: Table S3

Model 1 was unadjusted; Model 2 was adjusted for age and sex; Model 3 was adjusted for age, sex and vascular risk factors (including cigarette smoking, alcohol consumption, hypertension, diabetes mellitus, dyslipidemia, hyperhomocysteinemia, previous ischemic stroke and coronary heart disease); Model 4 was adjusted for age, sex, vascular risk factors and clinical data (including SBP, DBP, HR, blood glucose, admission NIHSS, onset-to-alteplase bolus time, TOAST, infarct location, and bridging therapy); Model 5 was adjusted using the confounders of Model 4 except for admission NIHSS score

*Abbreviations*: *OR* Odds ratio, *CI* Confidence interval, *SBP* Systolic blood pressure, *DBP* Diastolic blood pressure, *HR* Heart rate, *NIHSS* National Institutes of Health Stroke Scale, *TOAST* Trial of Org 10,172 in Acute Stroke Treatment classification, *mRS* modified Rankin Scale

^a^The NIHSS score at 24 h was determined for all 1072 patients, of which 493 had dominant hemisphere stroke and 488 had non-dominant hemisphere stroke

^b^Magnetic resonance imaging was available for 986 patients. Therefore, 986 patients were included in the analysis of infarct volume, of whom 454 had dominant hemisphere stroke and 449 had non-dominant hemisphere stroke

^c^All 1072 patients were included in the analysis of functional outcomes assessed by mRS, of whom 594 had mRS 2–6 (301 had dominant hemisphere stroke and 241 had non-dominant hemisphere stroke)

### Serum S100β levels and functional indicators

As shown in Additional file 1: Table S2, in univariable and multivariable linear regression, the highest S100β levels (> 0.20 ng/mL) were independently associated with higher 24-h and 7-day NIHSS scores. We also observed a significant interaction between stroke lateralization (dominant, non-dominant, and both hemispheres) and NIHSS score measured at 24 h after IVT (*P*_interaction_ < 0.001). In patients with dominant hemisphere stroke, the highest S100β levels (> 0.20 ng/mL) were associated with higher NIHSS score; however, no significant association was found in patients with non-dominant hemisphere stroke (Table 2 and Additional file 1: Table S3). Further, as depicted in Fig. 2B, the association between 24-h NIHSS score and S100β levels was more remarkable in patients with dominant hemisphere stroke than those with non-dominant hemisphere stroke.

**Fig. 3:**
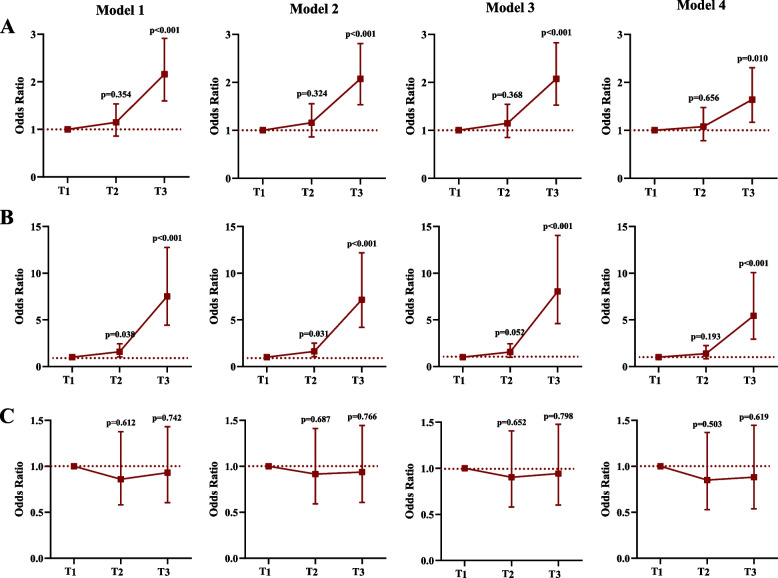
Association between S100β tertiles and unfavorable outcome at 90 days according to different models of **A** all patients, **B** patients with dominant hemisphere stroke, and **C** patients with non-dominant hemisphere stroke. Notes: Model 1 was unadjusted; Model 2 was adjusted for age and sex; Model 3 was adjusted for age, sex and vascular risk factors (including cigarette smoking, alcohol consumption, hypertension, diabetes mellitus, dyslipidemia, hyperhomocysteinemia, previous ischemic stroke and coronary heart disease); Model 4 was adjusted for age, sex, vascular risk factors and clinical data (including SBP, DBP, HR, blood glucose, admission NIHSS, onset-to-alteplase bolus time, TOAST, infarct location, and bridging therapy). Abbreviations: SBP, systolic blood pressure; DBP, diastolic blood pressure; HR, heart rate; NIHSS, National Institutes of Health Stroke Scale; TOAST, Trial of Org 10,172 in Acute Stroke Treatment classification

For functional outcomes assessed using the mRS score, S100β levels were significantly increased in patients with unfavorable outcomes compared with those with favorable outcomes (0.140 (0.062–0.393) ng/mL vs. 0.100 (0.050–0.196) ng/mL, *P* < 0.001). Univariable and multivariable binary logistic regression analyses revealed an independent association between S100β levels and unfavorable outcomes (Additional file 1: Table S2 and Fig. 3A). Moreover, the highest S100β level (> 0.20 ng/mL) was an independent predictive factor for all-cause death within 3 months of IVT (Additional file 1: Table S2).

The heterogeneity in stroke lateralization in the prognostic function (favorable outcome) of S100β was observed (*P*_interaction_ < 0.001). In patients with dominant hemisphere stroke, the highest S100β levels (> 0.20 ng/mL) were independently associated with unfavorable outcomes at 3 months, and patients with the highest S100β levels (> 0.20 ng/mL) 24 h after IVT presented a 4.4-fold increase in the risk of unfavorable 3-month outcomes compared with those with the lowest S100β levels. However, no significant association was found in patients with non-dominant hemisphere stroke (Table 2, Additional file 1: Table S3 and Fig. 3B, C). The distribution of functional outcomes at 90 days according to S100β tertiles in all patients, patients with dominant hemisphere stroke, and those with non-dominant hemisphere stroke is shown in Fig. 2C.

## Discussion

The present study showed that serum S100β levels 24 h after IVT were independently associated with HT, infarct volume, and prognosis in stroke patients who received IVT. Because the detection of S100β is convenient and has guiding significance for the formulation of clinical diagnosis and treatment strategy, it has great potential in clinical application.

HT is a major complication of IVT treatment, and reliable predictors remain limited. After brain tissue infarction, S100β levels are significantly elevated in the brain and enter the peripheral blood via the damaged blood–brain barrier [6, 18]. Thus, the level of S100β in the blood can partly reflect the extent of damage to the blood–brain barrier [18], suggesting that S100β is associated with HT following IVT. Foerch et al. reported that a S100β value in the highest quintile before thrombolytic therapy was independently associated with any HT [19]. In our study, the highest S100β levels 24 h after IVT were also independently correlated with HT. This suggests that S100β can assist in the assessment of HT both before and after thrombolysis, providing more methods for clinicians to indicate HT.

The prognostic value of functional outcomes based on S100β has been well demonstrated in patients with traumatic brain injury [20–22]. Recently, S100β levels in patients with acute ischemic stroke have been studied. Branco et al. demonstrated that serum S100β levels can potentially predict the 3-month prognosis of stroke patients [9]. However, the association of serum S100β levels with prognosis in stroke patients who received IVT remains unclear. In our multicenter study with a relatively large sample size, we found that the highest S100β level was both independently correlated with short-term (7-day NIHSS score) and long-term (all-cause death at 3 months and 3-month mRS) prognosis. Therefore, serum S100β levels may be used as an early biomarker to predict outcomes in patients with stroke.

In addition, we also found a result worth exploring in this study: S100β level was similarly associated with infarct volume in patients with stroke in dominant and non-dominant hemispheres, but it was only independently associated with 24-h higher NIHSS score and 3-month unfavorable outcomes in patients with dominant hemisphere stroke but the association was not significant in those with non-dominant hemisphere stroke. This may be because S100β level is only related to infarction volume, not infarction location, while the neurological scores (e.g., NIHSS and mRS scores) are affected by location [23–25]. The neurological scores emphasize deficits associated with lesions located in the dominant hemisphere (such as language or dominant hand function), and thus dominant hemisphere stroke is usually associated with higher scale scores [23]. Nevertheless, stroke in the non-dominant hemisphere, which is mainly associated with neglect, cognitive deficit, or apraxia, cannot be adequately reflected by stroke scales and is poorly detected by outcome examiners or patients’ relatives but may significantly influence a patient’s quality of life [24]. Our findings are not isolated. A previous study involving patients after mechanical thrombectomy found similar results: the correlation between infarct volume and functional outcome of patients with infarcts in the right (non-dominant) hemisphere was not as strong as that in patients with left (dominant) hemisphere infarct [25]. Our results, together with previous results, suggest that serum S100β level may be an important marker to assist in determining stroke severity regardless of infarct location. Furthermore, because these scales and S100β each have their own advantages, we speculated that finding different weighted coefficients of scale scores and S100β and combining them to predict a more accurate prognosis for patients after IVT will be a future direction of research.

The S100β detection technique used in this study is also worth popularizing. Traditionally, the quantitative assessment of S100β is performed with an enzyme-linked immunosorbent assay [9, 26, 27], which is manual and requires several hours, limiting the clinical usefulness of S100β as a biomarker. In our study, we used an automated bedside rapid testing technique, a magnetic particle-based chemiluminescent enzyme immunoassay analysis system. Quantitative S100β concentrations can be obtained within 28 min with high sensitivity, specificity, and reproducibility [28]. This convenient and rapid detection method is conducive to the promotion and application of our research results in clinical diagnosis and treatment.

Our study has some limitations. First, the number of patients receiving bridging therapy was limited; therefore, subgroup analysis could not be performed. Further studies remain warranted to explore the association between S100β levels and clinical outcomes in patients undergoing bridging therapy or mechanical thrombectomy. Second, due to the impact of COVID-19, many blood samples could not be collected due to a lack of transportation. Third, as shown in the footnote of Table 1, 86 (8.02%) of the patients did not undergo MRI examination, moreover, the lower the S100β level, the more cases were missing (T1: 10.39%; T2: 7.65%; T3: 5.76%). We considered that this was likely because patients with lower S100β levels may not be willing to undergo further MRI examination for milder disease to avoid higher hospitalization costs. These missing data limited the robustness of our conclusion. Further studies are warranted. Fourth, two parameters, the 24-h NIHSS and HT, were measured at the same time as serum S100β, thus the reported associations of S100β levels and these two parameters cannot be used for causal inference.

## Conclusions

Serum S100β levels 24 h after IVT were independently associated with HT, infarct volume, and prognosis in patients with IVT, which suggests the application value of serum S100β in judging the degree of patients’ disease and predicting prognosis.

## Supplementary Information

Supplementary Material 1. Fig. S1 and Tables S1-3. Fig. S1. Flowchart of the study; Table S1. Sixteen hospitals in the study; Table S2. The association of tertiles of S100β with clinical parameters and outcome indicators in total cohort; Table S3. The association of tertiles of S100β with clinical parameters and outcome indicators in patients with different lateralization stroke.

## Data Availability

The data used and/or analyzed during the current study are available in the manuscript and Additional files, except for the values of outcome measures that could be available only from the corresponding author on reasonable request.

## Abbreviations

CI: Confidence interval
ECASS: European Collaborative Acute Stroke Study
HI1: Hemorrhagic infarction type 1
HI2: Hemorrhagic infarction type 2
HT: Hemorrhagic transformation
IVT: Intravenous thrombolysis
mRS: Modified Rankin Scale
NIHSS: National Institutes of Health Stroke Scale
OR: Odds ratio
PH1: Parenchymal hematoma type 1
PH2: Parenchymal hematoma type 2
rPH: Remote parenchymal hematoma
TOAST: Trial of Org 10172 in the Acute Stroke Treatment
