# Supplementary material for: The association between serum S100β levels and prognosis in acute stroke patients after intravenous thrombolysis: a multicenter prospective cohort study
Source: BMC Med. 2024 Oct 3;22:304. doi: 10.1186/s12916-024-03517-6 (PMC11447957; doi:10.1186/s12916-024-03517-6)
Supplement: Supplementary file 1 — Supplementary Material 1. Fig. S1 and Tables S1-3. Fig. S1. Flowchart of the study; Table S1. Sixteen hospitals in the study; Table S2. The association of tertiles of S100β with clinical parameters and outcome indicators in total cohort; Table S3. The association of tertiles of S100β with clinical parameters and outcome indicators in patients with different lateralization stroke. [file 12916_2024_3517_MOESM1_ESM.docx]

**
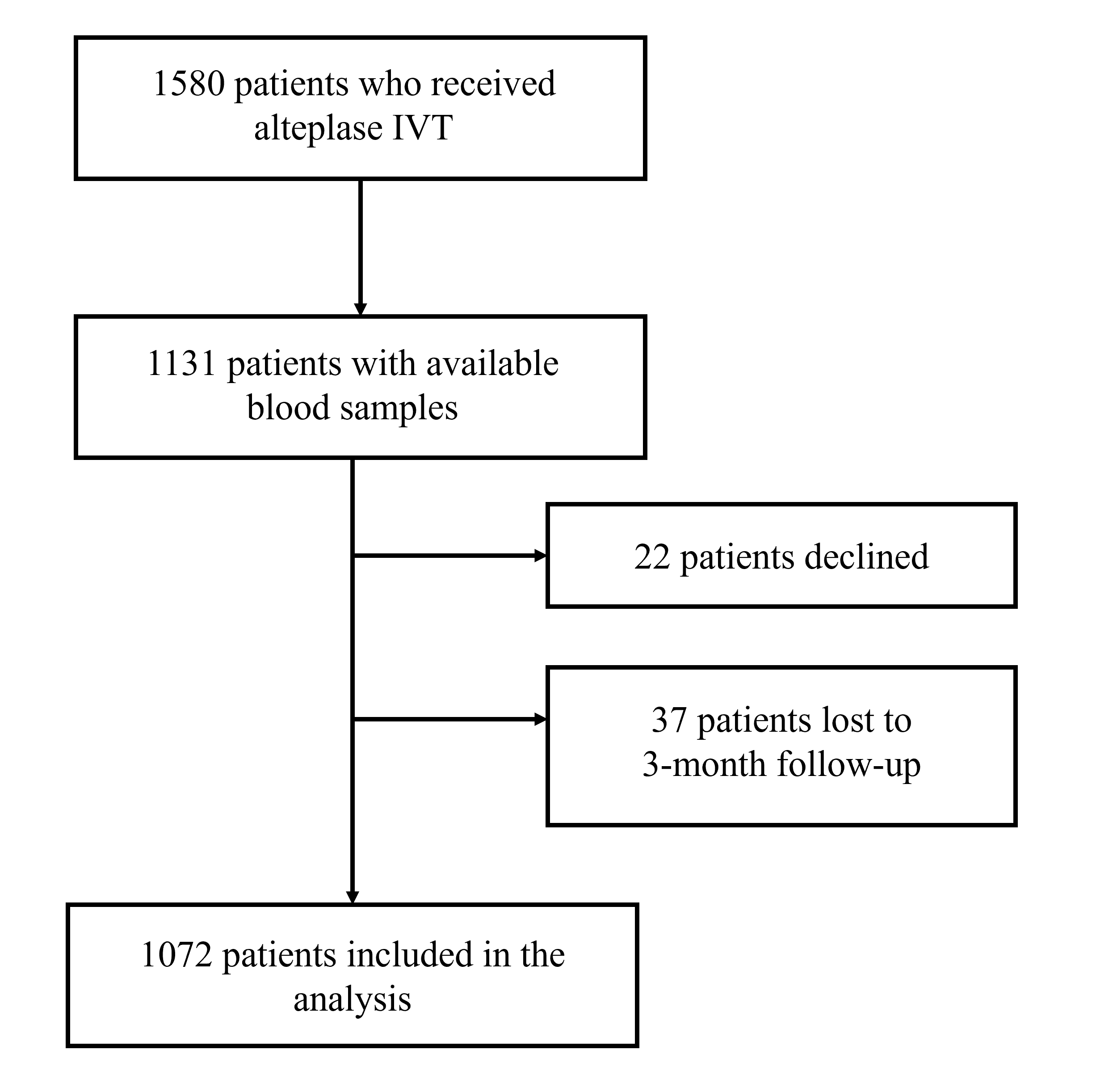
**

**Fig. S1. Flowchart of the study.**

Notes: Of the 1580 patients who received alteplase IVT, blood samples were collected from 1131; 22 declined to participate in the study during follow-up, 37 were lost to 3-month follow-up, and the remaining 1072 were included in the final analysis, of whom 2 patients had missing CT data and 1070 patients were included in the analysis of HT, 86 patients had missing MRI data and 986 patients were included in the analysis of infarct volume, 16 patients had missing 7-day NIHSS data and 1056 patients were included in the analysis of 7-day NIHSS, and all of 1072 patients were included in the analysis of 24-hour NIHSS and 3-month mRS.

Abbreviation: IVT=intravenous thrombolysis; CT=computed tomography; HT=hemorrhagic transformation; MRI=Magnetic resonance imaging; NIHSS=National Institutes of Health Stroke Scale; mRS=modified Rankin Scale.

**Table S1. Sixteen hospitals in the study.**

| **City** | **Hospital** |
| --- | --- |
| Changchun City | The First Hospital of Jilin University |
|  | Jilin Province People’s Hospital |
|  | Changchun People's Hospital |
|  | Dehuishi People's Hospital |
| Jilin City | Jilin City Hospital of Chemical Industry |
|  | Jilin Central General Hospital |
|  | Jilin People's Hospital |
|  | Affiliated Hospital of Jilin Medical College |
| Siping City | Siping Central People's Hospital |
|  | Jilin Neuropsychiatric Hospital |
| Tonghua City | Tonghua City Vascular Disease Hospital and Dongchang District People's Hospital |
| Songyuan City | Songyuan Jilin Oilfield Hospital |
|  | Songyuan Central Hospital |
|  | Qianguoerros Mongolian Autonomous County Hospital |
|  | Songyuan Hospital of Integrated Traditional Chinese and Western Medicine |
| Liaoyuan City | Dongliao First People's Hospital |

**Table S2. The association of tertiles of S100β with clinical parameters and outcome indicators in total cohort.**

|  | **Model 1** | | **Model 2** | | **Model 3** | | **Model 4** | | **Model 5** |  |
| --- | --- | --- | --- | --- | --- | --- | --- | --- | --- | --- |
|  | **OR/β (95%CI)** | ***P*** | **OR/β (95%CI)** | ***P*** | **OR/β (95%CI)** | ***P*** | **OR/β (95%CI)** | ***P*** | **OR/β (95%CI)** | ***P*** |
| **HT*** | | | | | | | | |  |  |
| HI1+HI2+PH1+PH2+rPH (Odds ratio) | | | | | | | | |  |  |
| T1 | Reference |  | Reference |  | Reference |  | Reference |  | Reference |  |
| T2 | 1.208 (0.633-2.303) | 0.567 | 1.210 (0.634-2.309) | 0.562 | 1.246 (0.651-2.385) | 0.507 | 1.211 (0.628-2.335) | 0.567 | 1.219 (0.633-2.347) | 0.554 |
| T3 | 3.562 (2.066-6.143) | ***<0.001*** | 3.480 (2.014-6.013) | ***<0.001*** | 3.680 (2.108-6.425) | ***<0.001*** | 3.126 (1.749-5.587) | ***<0.001*** | 3.407 (1.919-6.049) | ***<0.001*** |
| *P* for trend | ***<0.001*** |  | ***<0.001*** |  | ***<0.001*** |  | ***<0.001*** |  | ***<0.001*** |  |
| HI1+HI2+PH1+PH2 (Odds ratio) | | | | | | | | |  |  |
| T1 | Reference |  | Reference |  | Reference |  | Reference |  | Reference |  |
| T2 | 1.143 (0.585-2.235) | 0.695 | 1.145 (0.586-2.239) | 0.692 | 1.177 (0.600-2.308) | 0.636 | 1.135 (0.573-2.248) | 0.716 | 1.144 (0.579-2.261) | 0.699 |
| T3 | 3.688 (2.114-6.433) | ***<0.001*** | 3.614 (2.068-6.316) | ***<0.001*** | 3.878 (2.195-6.850) | ***<0.001*** | 3.292 (1.816-5.968) | ***<0.001*** | 3.620 (2.011-6.514) | ***<0.001*** |
| *P* for trend | ***<0.001*** |  | ***<0.001*** |  | ***<0.001*** |  | ***<0.001*** |  | ***<0.001*** |  |
| **Infarct volume† (Beta coefficient)** | | | | | | | | |  |  |
| T1 | Reference |  | Reference |  | Reference |  | Reference |  | Reference |  |
| T2 | 1,652 (-6.991 to 10.296) | 0.708 | 1.641 (-7.000 to 10.283) | 0.709 | 1.664 (-6.991 to 10.319) | 0.706 | 1.081 (-7.442 to 9.604) | 0.803 | 1.299 (-7.375 to 9.974) | 0.769 |
| T3 | 32.227 (23.674-40.781) | ***<0.001*** | 31.579 (22.992-40.166) | ***<0.001*** | 32.168 (23.465-40.871) | ***<0.001*** | 27.781 (18.948-36.615) | ***<0.001*** | 31.534 (22.632-40.437) | ***<0.001*** |
| *P* for trend | ***<0.001*** |  | ***<0.001*** |  | ***<0.001*** |  | ***<0.001*** |  | ***<0.001*** |  |
| **NIHSS 24 hours‡ (Beta coefficient)** | | | | | | | | |  |  |
| T1 | Reference |  | Reference |  | Reference |  | Reference |  | Reference |  |
| T2 | 0.738 (-0.097 to 1.572) | 0.083 | 0.752 (-0.078 to 1.581) | 0.076 | 0.693 (-0.137 to 1.524) | 0.102 | 0.405 (-0.266 to 1.076) | 0.237 | 0.510 (-0.309 to 1.330) | 0.222 |
| T3 | 3.471 (2.641-4.301) | ***<0.001*** | 3.326 (2.498-4.154) | ***<0.001*** | 3.387 (2.549-4.225) | ***<0.001*** | 1.802 (1.103-2.501) | ***<0.001*** | 2.945 (2.101-3.789) | ***<0.001*** |
| *P* for trend | ***<0.001*** |  | ***<0.001*** |  | ***<0.001*** |  | ***<0.001*** |  | ***<0.001*** |  |
| **NIHSS at 7 days# (Beta coefficient)** | | | | | | | | |  |  |
| T1 | Reference |  | Reference |  | Reference |  | Reference |  | Reference |  |
| T2 | 0.684 (-0.132 to 1.499) | 0.101 | 0.694 (-0.117 to 1.506) | 0.094 | 0.684 (-0.131 to 1.499) | 0.100 | 0.447 (-0.264 to 1.158) | 0.218 | 0.553 (-0.256 to 1.362) | 0.180 |
| T3 | 3.333 (2.516-4.151) | ***<0.001*** | 3.219 (2.404-4.035) | ***<0.001*** | 3.295 (2.468-4.122) | ***<0.001*** | 2.079 (1.337-2.820) | ***<0.001*** | 2.921 (2.084-3.758) | ***<0.001*** |
| *P* for trend | ***<0.001*** |  | ***<0.001*** |  | ***<0.001*** |  | ***<0.001*** |  | ***<0.001*** |  |
| **mRS 2-6^$^ (Odds ratio)** | | | | | | | | |  |  |
| T1 | Reference |  | Reference |  | Reference |  | Reference |  | Reference |  |
| T2 | 1.148 (0.858-1.537) | 0.354 | 1.160 (0.864-1.556) | 0.324 | 1.146 (0.852-1.543) | 0.368 | 1.075 (0.783-1.475) | 0.656 | 1.115 (0.824-1.507) | 0.480 |
| T3 | 2.158 (1.599-2.913) | ***<0.001*** | 2.074 (1.533-2.806) | ***<0.001*** | 2.075 (1.524-2.825) | ***<0.001*** | 1.642 (1.170-2.304) | ***<0.001*** | 1.937 (1.406-2.667) | ***<0.001*** |
| *P* for trend | ***<0.001*** |  | ***<0.001*** |  | ***<0.001*** |  | ***0.005*** |  | ***<0.001*** |  |
| **Death^$^ (mRS 6, Odds ratio)** | | | | | | | | |  |  |
| T1 | Reference |  | Reference |  | Reference |  | Reference |  | Reference |  |
| T2 | 2.112 (0.773-5.773) | 0.145 | 2.090 (0.761-5.740) | 0.153 | 1.962 (0.708-5.432) | 0.195 | 2.133 (0.733-6.209) | 0.165 | 1.951 (0.700-5.440) | 0.201 |
| T3 | 7.086 (2.942-17.065) | ***<0.001*** | 6.338 (2.617-15.348) | ***<0.001*** | 6.152 (2.511-15.073) | ***<0.001*** | 4.405 (1.685-11.510) | ***0.002*** | 5.741 (2.305-14.298) | ***<0.001*** |
| *P* for trend | ***<0.001*** |  | ***<0.001*** |  | ***<0.001*** |  | ***<0.001*** |  | ***<0.001*** |  |

Notes:

Model 1 was unadjusted; Model 2 was adjusted for age and sex; Model 3 was adjusted for age, sex and vascular risk factors (including cigarette smoking, alcohol consumption, hypertension, diabetes mellitus, dyslipidemia, hyperhomocysteinemia, previous ischemic stroke and coronary heart disease); Model 4 was adjusted for age, sex, vascular risk factors and clinical data (including SBP, DBP, HR, blood glucose, admission NIHSS, onset-to-alteplase bolus time, TOAST, infarct location and bridging therapy); Model 5 was adjusted using the confounders of Model 4 except for admission NIHSS score.

* Computed tomography scans at 24 hours were available for 1070 patients (no HT=977; HI1=27; HI2=21; PH1=20; PH2=21; rPH=4). Therefore, 1070 patients were included in the HT regression models.

‡ The NIHSS score at 24 hours was determined for all 1072 patients.

† Magnetic resonance imaging were available for 986 patients. Therefore, 986 patients were included in the analysis of infract volume.

# The NIHSS scores at 7 days were available for 1056 patients because 16 patients died or discharge within 7 days.

$ All 1072 patients were included in the analysis of functional outcomes assessed by mRS, of which 594 had mRS 2-6 and 52 were dead.

Abbreviations: HT=hemorrhagic transformation; OR=odds ratio; CI= confidence interval; HI=hemorrhagic infarction; PH=parenchymal hematoma; rPH=remote parenchymal hematoma; NIHSS=National Institutes of Health Stroke Scale; SBP=systolic blood pressure; DBP=diastolic blood pressure; HR=heart rate; TOAST=Trial of Org 10172 in Acute Stroke Treatment classification; mRS=modified Rankin Scale.

**Table S3. The association of tertiles of S100β with clinical parameters and outcome indicators in patients with different lateralization stroke.**

|  | **Model 5** | |
| --- | --- | --- |
|  | **OR/β (95%CI)** | ***P*** |
| **NIHSS 24 hours‡** (Beta coefficient) |  |  |
| Dominant hemisphere |  |  |
| T1 | Reference |  |
| T2 | 1.203 (-0.006 to 2.411) | 0.051 |
| T3 | 5.942 (4.665-7.219) | ***<0.001*** |
| *P* for trend | ***<0.001*** |  |
| Non-dominant hemisphere |  |  |
| T1 | Reference |  |
| T2 | -0.359 (-1.548 to 0.830) | 0.553 |
| T3 | 0.237 (-0.983 to 1.457) | 0.702 |
| *P* for trend | 0.738 |  |
| **Infract volume†** (Beta coefficient) |  |  |
| Dominant hemisphere |  |  |
| T1 | Reference |  |
| T2 | -1.633 (-15.060 to 11.795) | 0.811 |
| T3 | 46.995 (33.042-60.948) | ***<0.001*** |
| *P* for trend | ***<0.001*** |  |
| Non-dominant hemisphere |  |  |
| T1 | Reference |  |
| T2 | 6.003 (-6.467 to 18.472) | 0.345 |
| T3 | 23.134 (10.145-36.123) | ***<0.001*** |
| *P* for trend | ***<0.001*** |  |
| **mRS 2-6**$ (Odds ratio) |  |  |
| Dominant hemisphere |  |  |
| T1 | Reference |  |
| T2 | 1.562 (0.985-2.478) | 0.058 |
| T3 | 7.617 (4.252-13.646) | ***<0.001*** |
| *P* for trend | ***<0.001*** |  |
| Non-dominant hemisphere |  |  |
| T1 | Reference |  |
| T2 | 0.817 (0.518-1.287) | 0.383 |
| T3 | 0.886 (0.555-1.414) | 0.612 |
| *P* for trend | 0.583 |  |

Notes: The number of patients with both sides of stroke was limited, therefore, the analysis was conducted in patients with dominant or non- dominant hemisphere stroke.

Model 1 was unadjusted; Model 2 was adjusted for age and sex; Model 3 was adjusted for age, sex and vascular risk factors (including cigarette smoking, alcohol consumption, hypertension, diabetes mellitus, dyslipidemia, hyperhomocysteinemia, previous ischemic stroke and coronary heart disease); Model 4 was adjusted for age, sex, vascular risk factors and clinical data (including SBP, DBP, HR, blood glucose, admission NIHSS, onset-to-alteplase bolus time, TOAST, infarct location and bridging therapy). Model 5 was adjusted using the confounders of Model 4 except for admission NIHSS score.

‡ The NIHSS score at 24 hours was determined for all 1072 patients, of which 493 had dominant hemisphere stroke and 488 had non-dominant hemisphere stroke.

† Magnetic resonance imaging was available for 986 patients. Therefore, 986 patients were included in the analysis of infarct volume, of whom 454 had dominant hemisphere stroke and 449 had non-dominant hemisphere stroke.

$ All 1072 patients were included in the analysis of functional outcomes assessed by mRS, of whom 594 had mRS 2-6 (301 had dominant hemisphere stroke and 241 had non-dominant hemisphere stroke).

Abbreviations: OR=odds ratio; CI=confidence interval; SBP=systolic blood pressure; DBP=diastolic blood pressure; HR=heart rate; NIHSS=National Institutes of Health Stroke Scale; TOAST=Trial of Org 10172 in Acute Stroke Treatment classification; mRS=modified Rankin Scale.
